# The Effects of α-Tocopherol on Bone: A Double-Edged Sword?

**DOI:** 10.3390/nu6041424

**Published:** 2014-04-10

**Authors:** Kok-Yong Chin, Soelaiman Ima-Nirwana

**Affiliations:** Department of Pharmacology, Faculty of Medicine, Universiti Kebangsaan Malaysia, Jalan Raja Muda Abdul Aziz, Kuala Lumpur 50300, Malaysia; E-Mail: gabrielyong@live.com.my

**Keywords:** α-tocopherol, bone, fracture, osteoporosis, vitamin E

## Abstract

Recent studies have found conflicting evidence on the role of α-tocopherol (αTF) on bone health. This nonsystematic review aimed to summarize the current evidence on the effects of αTF on bone health from cell culture, animal, and human studies in order to clarify the role of αTF on bone health. Our review found that αTF exerted beneficial, harmful or null effects on bone formation cells. Animal studies generally showed positive effects of αTF supplementation on bone in various models of osteoporosis. However, high-dose αTF was possibly detrimental to bone in normal animals. Human studies mostly demonstrated a positive relationship between αTF, as assessed using high performance liquid chromatography and/or dietary questionnaire, and bone health, as assessed using bone mineral density and/or fracture incidence. Three possible reasons high dosage of αTF can be detrimental to bone include its interference with Vitamin K function on bone, the blocking of the entry of other Vitamin E isomers beneficial to bone, and the role of αTF as a prooxidant. However, these adverse effects have not been shown in human studies. In conclusion, αTF may have a dual role in bone health, whereby in the appropriate doses it is beneficial but in high doses it may be harmful to bone.

## 1. Introduction

Our skeletal system is tightly regulated by a multitude of internal and external factors, which govern bone formation and resorption. Osteoporosis represents a classic example of this regulation gone astray, where bone resorption occurs at a higher rate than bone formation. This results in fragile bone due to degenerative changes in the microarchitecture of bone tissue and a reduction in bone mass [1]. The World Health Organization defines osteoporosis as bone mineral density (BMD), as measured by dual-X ray absorptiometry, lower than 2.5 standard deviations (SD) from the young adult mean value (*T* score < −2.5 SD) [2]. Post-menopausal women are at greater risk for osteoporosis due to estrogen deficiency. Men also suffer from osteoporosis but it occurs at a later stage of their life [3,4,5]. Common risk factors for osteoporosis are low peak bone mass, low body weight, the use of certain medications (glucocorticoids, anticonvulsants, lithium, *etc.*), low Vitamin D and calcium intake, endocrine disorders (hypogonadism, hyperparathyroidism, hyperthyroidism, *etc.*), prolonged immobility, cigarette smoking, alcoholism and systemic inflammation [6,7].

There are other theories on the development of osteoporosis. One of the theories revolves around oxidative stress. Cross-sectional studies found that blood levels of oxidation products like 8-*iso*-prostaglandin F_2α_ (8-*iso*-PGF_2α_) were associated negatively with BMD while antioxidant levels were associated positively with BMD [8,9,10,11]. These have been confirmed by case-control studies, whereby osteoporotic subjects had a higher level of oxidants and a lower level of antioxidants compared to control subjects [12,13]. Animal studies showed that the differentiation of osteoclasts in nuclear factor-erythroid 2-related factor 2 knock-out mice, which had impaired oxidative status, was significantly promoted [14]. *In vitro* studies showed that the presence of free radical species or their products promoted osteoclastogenesis via up-regulation of receptor activator of nuclear factor κ-B ligand (RANKL) expression and signaling but suppressed the differentiation of osteoblasts [15,16,17]. Natural products possessing antioxidant activity have been shown to protect bone health [18,19,20].

Sex hormone deficiency has been linked to inflammation [21]. A study by Khosla *et al.* showed that withdrawal of estrogen or testosterone caused elevation in interleukin-1 (IL1), interleukin-6 (IL6) and tumor necrosis factor-α (TNFα) levels in elderly men and treatment with either testosterone or estrogen alone was able to suppress this increase [22]. Similarly, a cessation of ovarian function has also been linked to an increase in inflammatory cytokines in post-menopausal women [23]. Inflammatory cytokines have been shown to promote osteoclastogenesis and inhibit bone formation [24]. For example, TNFα has been shown to augment RANK-induced osteoclast differentiation but inhibit SMAD signaling essential in osteoblast differentiation via nuclear factor κ-B [25]. Anti-inflammatory substances have been shown to prevent bone loss in inflammation-induced osteoporosis in animal models [26,27,28].

**Figure 1:**
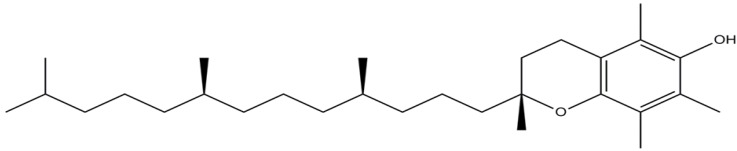
Molecular structure of α-tocopherol.

Large epidemiological studies have suggested that dietary components other than calcium are associated with bone health [29,30]. The role of vitamin E, a dietary component which functions as an antioxidant and antiinflammatory agent, in bone health have been studied but there are some conflicting findings [29,31]. Vitamin E consists of two major groups, which are tocopherols (TFs) and tocotrienols (TTs) [32,33]. The distinctive feature between these two groups is the presence of double bonds on the carbon chain of TTs [32,33]. There are four distinct isomers (α, β, γ and δ) in each group depending on the position of the methyl group on the chromanol ring [32,33]. Of all the isomers of vitamin E, α-tocopherol (αTF) is the most biologically relevant because of its abundance in nature and in health supplements, and also because it is selectively retained in our body through the action of αTF transporter protein (αTTP) in the liver (Figure 1) [34,35]. Previous reviews have discussed the effects of tocotrienols on bone health [36,37]. Therefore, the current discussion will focus on the relationship between αTF and bone health.

## 2. Literature Search

Literature search was performed using Scopus and Pubmed between November 2013 and January 2014. The search terms used were “α-tocopherol” AND “bone mineral density” OR “fracture risk” OR “bone remodeling” OR “osteoblast” OR “osteoclast” OR “osteoporosis”. Only literature written in English and dated since 1995 was included. Titles and abstracts of the selected articles were read by a reviewer and only the relevant literature was selected. Since we aimed to provide a scoping review on the effects of αTF on bone health, cell culture, animals, and human studies were considered. Intervention studies involving co-supplementation of αTF with other supplements were not included because it was impossible to dissociate the effects among the supplements on bone. Human observational studies that grouped the effects of αTF with other nutrients and did not distinguish the effects between each component were not included. For human studies, only those with valid bone health (bone mineral density or fracture risk assessment or bone remodeling markers) and αTF estimates (intake assessed by food frequency questionnaire or circulating level assessed by HPLC or both) were included. The final inclusion of the literature in the current review were discussed and agreed by the two authors. A systematic approach was not adopted in this review due to the heterogeneity of the human observational studies involved.

## 3. What did the *in Vitro* Evidence Show?

There are very limited *in vitro* studies that examine the relationship between αTF and bone health. The existing studies showed conflicting findings. Ahn *et al.* incubated human mesenchymal stem cells with αTF in an osteogenic differentiation media [38]. The results showed that these cells proliferated better in media with αTF. Treated cells also showed increased mRNA expressions of runt-related transcription factor 2 (RUNX-2) and transforming growth factor β1, as indicated by microarray analysis and real time reverse transcription-polymerase chain reaction. Since RUNX-2 has been shown to be an upstream regulator of osteoblastic genes, their results indicated that αTF could possibly promote osteoblastogenesis [38]. However, in a study by Urban *et al.* using primary bovine osteoblasts from the periosteum of calf metacarpus, the addition of 0.16 mg/mL αTF did not promote proliferation of osteoblasts [39]. α-TF also did not enhance the protein expression of collagen 1, osteonectin and osteocalcin as assessed via immunohistology [39]. On the other hand, Soeta *et al.* showed that TFs might suppress differentiation of osteoblasts isolated from rat calvariae [40]. α-TF (100 and 200 μM) and γTF (20 μM) decreased the mRNA expression of alkaline phosphatase significantly compared to untreated cells at the early stage of differentiation. The noncalcified nodule in treated cells in TF-treated cultures also appeared to be smaller compared to the untreated culture, which indicated a suppression of osteoid production. However, the size of the calcified nodule between the treated and untreated cells was not significantly different [40].

## 4. The Dual Nature of Vitamin E on Bone Health Revealed in Animal Studies

α-TF shows a plethora of biological activities, including prominent antioxidant and antiinflammatory activities [41,42,43]. Thus, the potential of αTF as an antiosteoporotic agent has been tested in animal studies. A study by Feresin *et al.* indicated that increases in osteoclast surface and eroded surface due to ovariectomy in aged female rats were prevented by supplementing them with 525 mg/kg diet (medium dose) and 750 mg/kg diet (high dose) of αTF for 100 days [44]. Mineralizing surface and bone formation rate were increased in rats supplemented with the medium dose of αTF. Rats supplemented with the high dose of αTF also showed increased biomechanical strength (ultimate and yield load, and ultimate and yield stress). However, all supplemented groups failed to demonstrate improvement in BMD and bone structural parameters. It should be noted that Feresin *et al.* induced osteopenia in these rats for 120 days before initiation of treatment. Hence, this experiment tested the ability of αTF to cure osteoporosis induced by estrogen deficiency in rats [44].

On the other hand, a series of studies were performed using a relatively low dose of αTF but a more consistent approach of supplementation (oral gavage) to study the effects of αTF on bone health in stressed rodents [45,46,47,48,49,50]. In these studies, the effects of αTF were compared to the unsupplemented stressed group and the tocotrienol-supplemented group [45,46,47,48,49,50]. Besides, treatment was initiated not long after ovariectomy (1–2 weeks post-surgery), so the ability of αTF to prevent osteoporosis induced by estrogen deficiency in rats was tested [45,46,47]. Using ovariectomized young rats, Muhammad *et al.* showed that αTF supplementation at 60 mg/kg body weight for four weeks prevented degenerative changes in trabecular bone structural parameters (bone volume, trabecular separation and number) [45]. Increase in osteoclast surface was also prevented. In this study, the efficacy of αTF was similar to palm TT mixture [45]. Nazrun *et al.* showed that αTF at 60 mg/kg body weight for eight weeks could marginally improve bone biomechanical parameters (maximum load and stress, stiffness and Young’s modulus) but the changes were not statistically significant [46]. α-TF at this dose was not able to improve oxidative status of these rats as shown by a persistent elevation of plasma malondialdehyde (MDA) level. In the same experiment, rats fed with palm TT mixture showed significantly lower MDA and higher antioxidant enzyme (superoxide dismutase and glutathione peroxidase) activity in plasma [46]. Norazlina *et al.* showed that αTF supplementation at 30 mg/kg body weight for 10 months preserved calcium content of the femur in ovariectomized rats [47]. It also increased alkaline phosphatase activity and reduced tartrate-resistant acid phosphatase activity in ovariectomized rats. In this study, the efficacy of αTF was higher compared to palm TT mixture at the same dose [47].

Apart from the ovariectomy model, the efficacy of αTF was tested in other osteoporosis rodent models. Hermizi *et al*. showed that αTF at 60 mg/kg body weight for two months was able to improve bone volume, trabecular number, mineral apposition rate and bone formation rate, and reduced osteoclast surface compared to nicotine-treated group [48]. However, the efficacy of αTF was lower compared to γTT and TT-enriched fraction [48]. Norazlina *et al.* attributed the protective effect of αTF to its ability to reduce inflammation elicited by nicotine treatment, shown by a reduction of IL1 level in the αTF-supplemented group [49]. Using an osteoporosis rat model induced by intraperitoneal injection of ferric nitrilotriacetate (an oxidizing agent), Ahmad *et al.* showed that all three doses (30, 60, 100 mg/kg body weight) of αTF were not able to protect the rats from degenerative changes in structural, cellular and dynamic parameters in bone [50]. In contrast, rats treated with palm TT mixture at 100 mg/kg body weight showed improvements in all of the parameters aforementioned [50].

The effects of αTF deficiency had been tested recently in two separate studies using genetically modified mice [51,52]. Fujita *et al.* showed that αTF transfer protein knock-out (αTTPKO) mice, which were not able to absorb vitamin E, had a higher bone mass compared to wild-type mice [51]. This phenotype was attributed to lower bone resorption in the αTTPKO mice, indicated by a lower osteoclast number and serum deoxypyridinoline level. This led to the speculation that Vitamin E played a part in regulating bone mass. *In vitro* experiments indicated that αTF promoted osteoclast differentiation and fusion without affecting the proliferation of its precursor [51]. This effect was shown exclusively by αTF but not with other vitamin E isomers, and it was not related to the antioxidant effect of αTF. They also showed that wild type mice fed with 600 mg/kg diet of αTF for eight weeks showed a 20% reduction in bone mass [51]. Iwaniec *et al*. attempted to validate the findings of Fujita *et al.* in aged αTTPKO mice [52]. They found that αTTPKO male mice had lower cross-sectional volume, cortical volume, and cortical thickness at the femoral diaphysis compared to wild type mice. No differences in trabecular bone parameters were observed. Furthermore, they showed that feeding wild type rats (8.5 months old) with diet containing 15 IU/kg, 75 IU/kg, 500 IU/kg diet of αTF for 13 weeks did not result in any changes in bone parameters [52]. The reasons for the differences in bone phenotype displayed by αTTPKO mice at different ages shown by these two studies are still unknown [51,52]. The negative effects of αTF on bone shown by Fujita *et al.* [51] could not be validated by Iwaneic *et al.* [52].

Other studies found that αTF in high doses could prevent osteoporosis in stress conditions but could be harmful to bone in normal conditions [53]. Smith *et al.* treated 8.5 month-old male rats which were ambulatory or subjected to hindlimb-unloading with 15 IU/kg, 75 IU/kg or 500 IU/kg diet of αTF [53]. They found that ambulatory animals supplemented with 500 IU/kg diet of αTF had a significantly lower trabecular number and bone volume compared to ambulatory animals supplemented with the lower doses of αTF [53]. However, supplementation of 500 IU/kg diet αTF successfully prevented osteoporosis induced by hindlimb-unloading [53]. The researcher attributed the bone protective of αTF effect to a lower expression of cyclooxygenase-2 in the supplemented group and thereby a reduction in inflammation [53]. However, the reduction in bone quality in the high-dose supplemented ambulatory group was not explained. In another study by Arjmandi *et al.*, young (six-month old) and aged (24-month old) mice were treated with 30 mg/kg diet or 500 mg/diet of αTF for 30 days [54]. The aged mice benefited from the high dose αTF treatment, as indicated by improved yield stress, ultimate load, yield load and stiffness of the bone. These changes were not observed in young mice. Despite this, the high-dose αTF increased osteocalcin and insulin-like growth factor-1 mRNA expression in both young and aged mice [54]. Both studies aforementioned could indicate that αTF would be beneficial to bone only if it was subjected to stress [53,54]. High dose of αTF might exert no effect on normal bone, or it might even be harmful.

## 5. The Association between Dietary αTF and Bone Health in Humans

Several epidemiological studies had been conducted to assess the effects of vitamin E on bone health [29,30,31,55,56,57,58,59,60,61]. These studies used either the blood level or the consumption level of αTF determined using food frequency questionnaires as the surrogate of total vitamin E [29,30,31,55,56,57,58,59,60,61]. This was a reasonable approach since αTF is the most abundant vitamin E isomer present in food and the most widely distributed in our body [34,35]. The term “vitamin E” was used in some studies but the measurements were indicative of αTF only [29,30,55,58,59,60]. Hence, we used “vitamin E (αTF)” when discussing these studies.

In a case control study, Maggio *et al.* compared the oxidative status of 75 osteoporotic (mean age = 70.4 ± 8.5 years) and 75 normal (mean age = 68.8 ± 3.5 years) post-menopausal women by measuring the enzymatic and non-enzymatic antioxidants in their blood [55]. Osteoporotic subjects were found to have significantly lower plasma vitamins A, C and E (αTF), and antioxidant enzymes superoxide dismutase and glutathione peroxidase. However, vitamin E was not correlated to femoral BMD in the osteoporotic subjects [55]. Since this study adopted a small sample size, its power in detecting an association between vitamin E and bone in the aforementioned subjects might be insufficient. The dietary records of the subjects were not collected; hence the intake of calcium and other antioxidant that might influence the results were not adjusted for.

In a Spanish post-menopausal population (*n* = 232 women; mean age = 56.9 ± 6.2 years), Mata-Granados *et al.* found that the ratio of serum αTF to lipid (αTF: lipid ratio) was significantly associated with BMD at the lumbar spine in both logistic and linear regression models [31]. This association was independent of age, body anthropometry, osteocalcin level, physical activity status, vitamin D level, smoking status and alcohol intake. The association between αTF:lipid ratio and BMD at the femoral neck was not significant. The researchers opined that this discrepancy might be due to the fact that the lumbar spine had more trabecular bone compared to the femoral neck [31]. While the sample size was moderate, this study was well-adjusted and the ratio of lipids to αTF was considered. Previous studies had shown that the absorption of αTF was higher when taken with a fat-rich meal [62].

Ostman *et al.* studied the associations of serum αTF level and oxidative stress at age 77 with BMD at age 82 in men (*n* = 405) [56]. They found that αTF alone did not explain the variation in BMD at all sites among the subjects. However, it modified the association between urinary 8-*iso*-PGF_2a_ level, a product of oxidative stress, and BMD (total body, proximal femur and lumbar spine). Subjects with low αTF and high 8-*iso*-PGF_2α_ had significantly lower BMD at all sites [56]. These long-lived subjects might represent a healthy selection, and might not be representative of the general population.

In the National Health and Nutrition Examination Survey, Hamidi *et al.* assessed the association between the serum levels of two vitamin E isomers: αTF and γTF and bone remodeling markers in 497 postmenopausal American women (mean age = 65.5 years with SE = 0.6 years) [57]. They found that a high serum αTF to γTF ratio was significantly and negatively associated with bone alkaline phosphatase (BAP) level. Furthermore, there was a decrease in BAP level with every increase in quintiles of αTF intake, serum αTF level and αTF to γTF ratio. A decrease in serum γTF level was also associated with a decrease in BAP level. The researchers also showed that supplementation of αTF reduced the serum γTF level. Hence, it was suggested that γTF might uncouple bone remodeling process by enhancing bone formation (as indicated by BAP level). α-TF might pose a threat to bone health by decreasing serum γTF level [57]. A major limitation of this study was that only one marker for bone formation and one marker for bone resorption were tested [57]. The testing of more markers for both bone remodeling phases could consolidate the findings of this study [57].

Two separate case-control studies determined the association between vitamin E (αTF) intake, smoking behavior and the risk of fracture [58,59]. Melhus *et al.* showed that in the Swedish Mammography Cohort (*n* case = 205, *n* control = 746, all post-menopausal women), current smokers with low intake of vitamin E assessed using food frequency questionnaire had a higher risk of suffering from a fracture, with an odds ratio of 3 [58]. The combination of low vitamin E, low vitamin C and current smoking further increased the odds ratio for fracture to 4.9. Similar interaction was not seen in other antioxidants studied [58]. In the Utah Study of Nutrition and Bone Health (*n* case = 1215, *n* control = 1349, men and women were involved), Zhang *et al.* showed that higher quintiles of vitamin E (αTF) intake was associated with lower prevalence of fractures in ever smokers (current and former smoker combined) but not in never smokers [59]. The association was inverse and linear [59]. These two studies illustrated again αTF might be protective to bone in stress conditions, by possibly acting as an antioxidant [58,59]. However, circulating αTF level was not available for both studies [58,59].

In two groups of young adult women in China (*n* = 441; age range: 20–35 years), Chan *et al.* observed that the intake of vitamin E (αTF) assessed using a food frequency questionnaire was positively correlated with total BMD at the spine, but not with total BMD at the hip and the femoral neck [60]. The study populations were from highly urbanized regions, and were not high risk for osteoporosis; hence they might not represent the general population.

MacDonald *et al.* performed a longitudinal study to determine the influence of dietary components, including vitamin E on BMD changes in a group of early menopausal women (*n* = 891 women; age range at baseline = 45–55 years; age range at follow-up = 50–59 years) using questionnaires [29]. They found that vitamin E intake (αTF) from diet alone was negatively associated with changes in BMD at the lumbar spine and femoral neck. However, total vitamin E intake including supplements was not associated significantly with changes in BMD. The researchers opined that the intake of dietary vitamin E was significantly confounded by the intake of polyunsaturated fatty acids, so the significance could be false and vitamin E might simply be a surrogate marker for fat intake [29].

A recent longitudinal study by Michaëlsson *et al.* examined the intake and serum concentration of αTF in relation to fractures in 61,422 elderly women from the Swedish Mammography Cohort (followed for 19 years) and 1138 elderly men (followed for 12 years) from the Uppsala Longitudinal Study of Adult Men [61]. The hazard ratio for hip fracture and any fracture increased linearly with decreasing dietary αTF intake for women. α-TF supplementation was also associated to decreased fractures rate in these women. The hazard ratio for fractures increased in men with lower quintiles of dietary αTF intake compared to men with the highest quintiles but the trend was not linear. An increase in serum αTF level was also associated with a decrease in fracture rate in men [61].

In the Women’s Health Initiative Observational Study and Clinical Trial (*n* = 11,068; age range = 50–79 years), Wolf *et al.* studied the association between the serum level and consumption level of Vitamin E (αTF) with BMD [30]. They observed that serum Vitamin E and other antioxidant levels were not associated with BMD at the femoral neck. The intake of vitamin E and all antioxidants was also not associated with BMD at the femoral neck after multiple adjustments [30].

Several limitations need to be considered in the interpretation of these human observational studies. Seven of the 10 studies mentioned above are cross-sectional studies [30,31,55,57,58,59,60]. Hence, they do not suggest any causal relationship between αTF and bone health. Four studies depend solely on dietary or food frequency questionnaire without circulating αTF measurement as the estimate of αTF level [29,58,59,60]. The use of questionnaire is subject to biases due to errors in reporting, especially self-administered questionnaire. The absorption of αTF depends on the food matrix consumed [63], genetic variability [64] and physiological condition of the subjects [62], hence αTF intake assessed by dietary questionnaire may not reflect the true circulating αTF level in the subject. The study by MacDonald *et al.* also suggested that αTF intake was highly confounded by fat intake [29]. This shows that it is difficult to separate the effect of one dietary component from another. Only four studies examine the relationship between αTF and bone as the primary objective [31,56,57,61], while most studies examine αTF as a part of the matrix of dietary component or antioxidant that might protect bone health. Due to the heterogeneity of the study designs and outcome measured, a systematic review was not attempted by the authors. The design of these human studies was summarized in Table 1.

## 6. Is the Relationship between αTF and Bone a U-Shape Curve?

**Figure 2:**
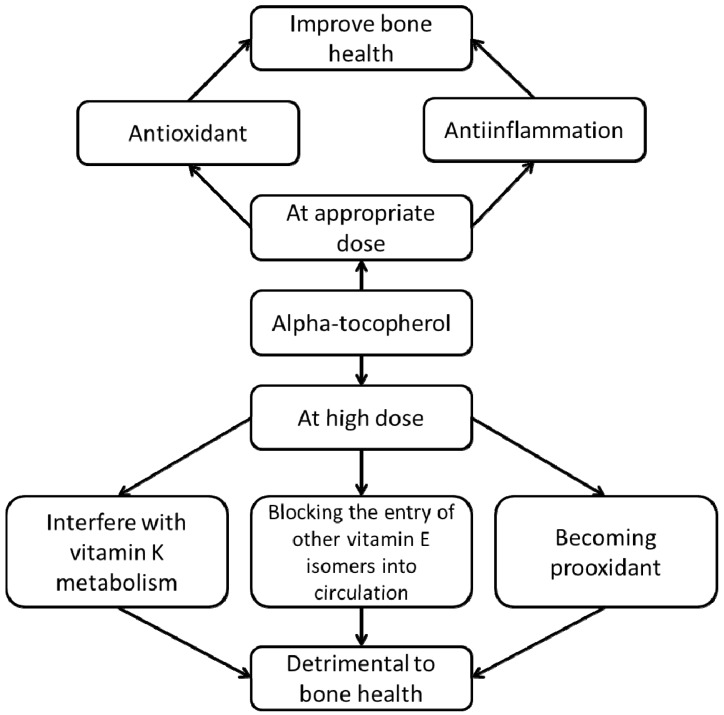
The proposed relationship between α-tocopherol and bone health.

Human observational studies generally show beneficial effects of αTF on bone health. Since most of the studies are dietary studies, the normal consumption of αTF in these populations are not exceedingly high. In animal studies, the effects of αTF supplementation differ between dosage and physiological condition, whereby in high doses αTF is potentially harmful to bone in normal animals. Based on the observations from animal studies, we propose that the relationship between αTF and bone health is U-shaped, whereby at lower doses it is protective but at higher doses it could exert adverse effects to bone. While the bone-sparing effects of αTF could be attributed to its antioxidant and antiinflammatory properties, its detrimental effects could be due to three factors, namely (1) the interaction between αTF and vitamin K, (2) the interaction between αTF and other vitamin E isomers, and (3) the prooxidant effects of αTF at high concentrations (Figure 2).

There has been speculation that αTF can interfere with the physiological function of vitamin K in the body [65]. Vitamin K is an essential factor in the coagulation cascade, and is also needed for the carboxylation of osteocalcin, which is an important non-collagenous protein in the bone matrix [66,67]. Previous studies showed that supplementation of αTF at high doses prolonged prothrombin time and increased hemorrhagic events in animals [68,69,70]. This coagulopathy could be reversed by the supplementation of vitamin K [69,70]. Booth *et al.* showed that supplementation of 1000 IU αTF for 12 weeks increased the degree of under-γ-carboxylation of prothrombin in healthy subjects and in subjects suffering from rheumatoid arthritis [71]. The degree of carboxylation of osteocalcin was not affected by αTF supplementation in this study [71]. Traber hypothesized that αTF could interfere with the physiological function of vitamin K by modifying xenobiotic pathways that facilitated its metabolism and excretion, and by competing with vitamin K enzymes essential for the conversion of phylloquinone (K1) to menaquinone-4 (MK-4), a more biologically active form of vitamin K [65]. Since vitamin K was found to be associated positively with BMD and negatively with fracture risk in human populations [72,73], enhanced vitamin K excretion or inhibition of its activation induced by high-dose αTF supplementation could be detrimental to bone health.

All isoforms of vitamin E are secreted by the liver into the blood stream by αTTP which exhibits distinct binding affinity to each isoform [74]. At high doses, αTF could compete effectively with other vitamin isoforms to bind with αTTP, thereby blocking their entry into the circulation. For example, the relative binding affinity of γTF to αTTP is only 8.9% of that of αTF [74]. Ikeda *et al.* showed that in rodents, co-administration of 50 mg/kg body weight αTT and 50 mg/kg body weight αTF significantly reduced the accumulation of αTT in blood and in various tissues [75]. Ha *et al.* demonstrated that mouse marrow macrophages incubated with αTT showed decrease formation of osteoclasts and resorption activity [76]. Handelman *et al.* showed that eight-week supplementation of 1200 IU αTF in humans significantly decreased their plasma γTF levels [77]. Huang *et al.* demonstrated that significant suppression of serum γTF could be achieved with αTF supplementation of 400 IU for eight weeks [78]. As indicated by Hamidi *et al.* in a cross-sectional study, γTF was related to bone formation markers and was hypothesized to uncouple bone remodeling in favor of bone formation [57]. The depletion of these beneficial vitamin E isoforms by high-dose αTF could be harmful to the skeletal system.

**Table 1:** The study designs of the human studies assessing the relationship between α-tocopherol and bone health.

No.	Authors (Year)	Study Design	Population/Sample Size	Method of Measurement	
				Vitamin E Intake	Bone Health
1	Melhus *et al*. 1999 [58]	Case-control (The Swedish Mammography Cohort)	205 cases, 746 controls. All postmenopausal women	Self-administered food frequency questionnaire (by mail)	Hip fracture incidence
2	Maggio *et al*. 2003 [55]	Case control	75 osteoporotic subject (age: 70.4 ± 8.5 years) and 75 normal control (age: 68.8 ± 3.5 years). All subjects are postmenopausal women	HPLC using αTF standard. Sample: Plasma	Bone health status (normal/osteoporotic) and femoral neck BMD
3	Macdonald *et al*. 2004 [29]	Longitudinal study	891 women aged 45–55 years at baseline and 50–59 years at follow up	Self-administered food frequency questionnaire (by mail)	Femoral neck and lumbar spine BMD
4	Wolf *et al*. 2005 [30]	Cross-sectional (Women’s Health Initiative Observational Study and Clinical Trial)	11,068 women aged 50–79 years	Dietary (self-administered) and supplementation (assisted) questionnaire and HPLC measuring αTG and γTF. Sample: Serum	Total body, lumbar spine, total hip (femoral neck and trochanter) BMD
5	Zhang *et al*. 2005 [59]	Case control/Retrospective (The Utah Study of Nutrition and Bone Health) Retrospective. Fracture happened then interviewed	Men and women. 1215 cases, 1349 control	Food frequency questionnaire	Hip fracture incidence
6	Ostman *et al*. 2009 [56]	Longitudinal (The Uppsala Longitudinal Study of Adult Men)	405 elderly men screened at 77 and 82 years	HPLC measuring αTF	Total body, proximal femur and lumbar spine BMD
7	Chan *et al*. 2009 [60]	Cross-sectional	221 women from Hong Kong and 220 women from Beijing. 20 to 35 years	Food frequency questionnaire (self-administered)	Total hip, femoral neck and total spine BMD
8	Hamidi *et al*. 2012 [57]	Cross-sectional. National Health and Nutrition Examination Survey	497 postmenopausal women, mean age 65.5 years (SE 0.6 years)	Food frequency questionnaire (24-h dietary recall) recorded by trained personnel and HPLC measuring αTF and γTF	Bone remodeling markers: serum alkaline phosphatase and urinary *N*-telopeptides
9	Mata-Granados *et al*. 2013 [31]	Cross-sectional	232 early postmenopausal Spanish Caucasian women (age: 56.9 ± 6.2 years) attending breast cancer screening	HPLC, vitamin E measured as αTF. Sample: Serum	Femoral neck and lumbar spine BMD
10	Michaëlsson *et al*. 2014 [61]	Longitudinal study (from the Swedish Mammography cohort and the Uppsala Longitudinal Study of Adult Men)	61,422 women (from SMC followed for 19 years) and 1138 men from (ULSAM followed for 12 years)	Food frequency questionnaire (self-administered) and HPLC	Hip and other fracture incidence

Although αTF is a prominent antioxidant, it is capable of acting as a prooxidant as well. Kontush *et al.* showed that in mild oxidative states and in the absence of other co-antioxidants like ascorbate, αTF behaved as a prooxidant by promoting the rate of lipid peroxidation in human plasma and low-density lipoprotein oxidation *in vitro*. [79]. They postulated that under the aforementioned circumstances, α-tocopheryl free radical could not form stable compounds by combining with another free radical or by donating the free electron to another antioxidant [79]. Therefore, it would interact with other macromolecules like lipids, thus generating more free radicals [79]. Considering these observations, it is reasonable to speculate that high-dose αTF supplementation could induce oxidative stress *in vivo* without adequate levels of other antioxidants. Hence, the oxidative stress generated could be harmful to the skeletal system.

The current evidence derived from human observational studies does not suggest αTF would increase fracture risk or decrease bone mineral density. The study by Hamidi *et al.* suggests a negative association of αTF level on bone formation but the long term effects is not known [57]. The adverse effects of αTF on bone are only evident in animal studies, whereby high-dose supplementation (500–600 IU/kg diet) decreases bone health of normal animals. It is questionable whether such high doses are achievable in human even with supplementation. Hence, it is not known the same adverse effects caused by high-dose αTF as observed in animals could happen in humans.

α-Tocopherol may have a dual role in bone health. At appropriate dosage it can be beneficial to bone by acting as an antioxidant countering oxidative stress, and as an antiinflammatory agent to reduce cytokines favorable to bone absorption. At high doses, it is hypothesized to interfere with vitamin K metabolism, to block the entry of other vitamin E isomers beneficial to bone into the circulation and to become prooxidant itself, thereby causing adverse effects on bone.

## 7. Conclusions

Our skeletal system is responsive to nutrients and supplements. α-TF, which possesses antioxidant and antiinflammatory effects, can be beneficial to bone health in appropriate doses. There is a lack of *in vitro* evidence on how αTF could influence bone health. Animal studies showed that αTF supplementation generally results in improved bone health as evaluated using bone histomorphometry and biomechanical strength. The effects were prominent in stress models, such as in osteoporosis induced by estrogen deficiency (ovariectomy), nicotine and hindlimb-unloading. In some studies, supplementation of high doses of αTF caused adverse effects on bone in healthy animals [53]. A few studies adopted genetically modified αTTP knock-out mice to study the effects of αTF on bone but the results were conflicting [51,52]. Cross-sectional studies in humans generally pointed to a positive association between blood αTF level and/or αTF consumption and bone health as assessed by BMD and fracture risk. Only one study showed a negative effect but this was confounded by other variables like the consumption of lipids [29]. We proposed that in stressed conditions and in appropriate doses, αTF exerts antiosteoporotic effects. However, in large doses, αTF could be harmful to bone via three possible mechanisms, including interference with Vitamin K metabolism and excretion, competitive binding for αTTP with other Vitamin E isomers beneficial to bone and thereby blocking their entry, and the prooxidant effects of αTF. These adverse effects have not been proven in human studies. These speculations have not been properly tested so far and should be examined closely to unveil the complex role of vitamin E in bone physiology. With the prevalence of αTF supplements in the market and the skyrocketing fracture incidence in developing countries, the need to solve this puzzle entails indispensable research efforts.
